# Supplementary material for: Factors related to a sense of economic insecurity among older adults who participate in social activities
Source: PLoS One. 2024 Mar 28;19(3):e0301280. doi: 10.1371/journal.pone.0301280 (PMC10977778; doi:10.1371/journal.pone.0301280)
Supplement: S2 Table — (PDF) [file pone.0301280.s002.pdf]

Table. Comparison between Analyzed and Excluded Participants n=872

	Analyzed (n=717)	Excluded (n=155)	p-value	effect size
**Basic attributes**				
Gender (n=866)				
Male	125 (17.4)	32 (21.5)	0.244	0.040
Female	592 (82.6)	117 (78.5)		
Age (n=861)				
65–74	186 (25.9)	28 (19.4)	0.002	0.122
75–84	404 (56.3)	72 (50.0)		
85＋	127 (17.7)	44 (30.6)		
Household composition (n=867)				
With spouse/children/others	480 (66.9)	108 (72.0)	0.228	0.041
Living alone	237 (33.1)	42 (28.0)		
Marital Status (n=863)				
Married	700 (98.0)	144 (96.6)	0.291	0.036
Unmarried	14 (2.0)	5 (3.4)		
Having a child (Children) (n=867)				
Yes	670 (93.4)	137 (91.3)	0.354	0.031
No	47 (6.6)	13 (8.7)		
Sense of economic insecurity (n=864)				
Not worried	404 (56.3)	86 (58.5)	0.631	0.016
Worried	313 (43.7)	61 (41.5)		
**Physical Conditions**				
Illness (n=865)				
No	105 (14.6)	19 (12.8)	0.568	0.019
Yes	612 (85.4)	129 (87.2)		
Subjective symptoms of dementia (n=857)				
No	649 (90.5)	113 (80.7)	＜0.001	0.115
Yes	68 (9.5)	27 (19.3)		
Useing long-term care insurance services (n=858)				
No	640 (89.3)	98 (69.5)	＜0.001	0.211
Yes	77 (10.7)	43 (30.5)		
**Social Conditions**				
Frequency of outing (n=856)				
Once a week＋	640 (89.3)	111 (79.9)	0.002	0.106
Less than once a week	77 (10.7)	28 (20.1)		
Frequency of participation in social activities (n=808)				
Once a week＋	609 (84.9)	77 (84.6)	0.936	0.003
Less than once a week	108 (15.1)	14 (15.4)		
Working Status (n=858)				
Yes	84 (11.8)	20 (13.5)	0.568	0.019
No	626 (88.2)	128 (86.5)		
Social Isolation（n=840）				
No	484 (67.5)	76 (61.8)	0.214	0.043
Yes	233 (32.5)	47 (38.2)		
**Psychological Conditions**				
Loneliness（n=856）				
Not lonely	637 (88.8)	114 (82.0)	0.025	0.077
Lonely	80 (11.2)	25 (18.0)		
Subjective Well-Being (n=855)	6.28 (2.74)	5.93 (2.82)	0.165	0.050

a：χ^2 test

b：The Philadelphia Geriatric Center Morale Scale, Mann-Whitney U test

Marital status and work were tested, excluding those who did not want to answer the question.

Data is presented as either n (%) or mean±standard deviation
