# Supplementary material for: Factors related to a sense of economic insecurity among older adults who participate in social activities
Source: PLoS One. 2024 Mar 28;19(3):e0301280. doi: 10.1371/journal.pone.0301280 (PMC10977778; doi:10.1371/journal.pone.0301280)
Supplement: Attachment — Submitted filename: renamed_1b3be.docx [file pone.0301280.s003.docx]

Responses to Reviewers’ Comments

Manuscript Number：PONE-D-23-12908

Full Title：Factors related to a sense of economic insecurity among older adults who participate in social activities

Dear. Reviewer #1

Thank you for giving us the opportunity to strengthen our manuscript with your valuable comments and queries.

1. The Abstract:

Delete the following sentence: This may be because the ....

Line 49: prevents → may prevent

**Reply:**

Thank you for your comment. We agree with you and have incorporated these suggestions (p. 2, lines 47–49).

2. INTRODUCTION: Frailty is a state of increased ... Although the Abstract did not mention any thing about frailty, the first paragraph of the Introduction mainly focuses on frailty. So, it is confusing whether the current manuscript targets only frail older adults. You may consider revising the Abstract OR revising the Introduction so that readers will not get confused.

**Reply:**

Thank you for your comment. We agree with you and have accordingly revised our paper.

Specifically, we have recast our expression in the Abstract (p. 2, lines 27–29) and Introduction (p. 3–4, lines 59–60,75–79,90–93) to clarify that the paper does not focus only on frail older adults.

3. Line 78: The top three causes and motives of suicide → Please describe the three causes and motives in detail.

**Reply:**

Thank you for this suggestion. In response, we have detailed the three causes and motives of suicide. The most common cause and motive of suicide among older adults is "health problems," followed by "family problems," and "economic and lifestyle problems (p. 3, lines 65–69).

4. Participants: who participated in social activities → this is a bit unclear. Please explain what social activities are you referring to and why you considered this group of older people to participate in the study. This should be also explained in the Introduction section so that readers would better understand the context of the data collection and the importance of the current study to this specific group of older adults.

**Reply:**

Thank you for your comment. The participants were older adults who participated in social activities that contributed to the prevention of long-term care, such as community-based exercise and hobby activities. The Ministry of Health, Labor and Welfare is working to expand these activities. We have added to and revised the Introduction (p. 4, lines 75–79) and Research Participants (p. 5, lines 100–103) sections to provide a better understanding of our research subjects and the importance of our research.

5. The survey was conducted from July to December 2022. → This means that the survey was conducted during the COVID-19 pandemic, where several social activities and group gathering were prohibited in may centers in Japan. This might have affected the available population, the response rate, and the results. Please elaborate on these issues in the Discussion.

**Reply:**

Thank you for your feedback. We agree with your assessment. The period from July to December 2022, when we conducted our study, was a period of increased infections due to the spread of BA.5, which was one of the more infectious Omicron strains. Accordingly, many social activities were carried out after ensuring infection prevention measures. In response to your excellent suggestion, we have explained our consideration of the effects of COVID-19 (p. 27–28, lines 344–356).

6. Line 122: Please delete this title (you already have a title for each category)

**Reply:**

　Thank you for your suggestion. We accordingly removed this title.

7. Please elaborate on the psychometric properties of all used instruments. For example, did you examine its reliability

of the outcome variable? If no, you need to explain why and how the results can be trusted in the absence of psychometric properties. Also, you need to elaborate on the available tools to assess economic insecurities in the literature.

**Reply:**

　　　Thank you for this query. We have ensured that this information is provided in the Materials and Methods section. We also describe the instruments we used below:

1. The self-administered dementia checklist:

The self-administered dementia checklist was developed as a 10-item and 2-factor instrument. Its factorial validity and internal reliability were confirmed. Cronbach’s α for the overall 10-item scale was 0.908. The concurrent and discriminant validities of the checklist were confirmed.

1. The Lubben Social Network Scale (LSNS-6):

The Lubben Social Network Scale (LSNS-6) is used worldwide as a screening tool for social isolation in older adults. The reliability and validity of the Japanese version of the LSNS-6 were confirmed. Cronbach’s α was 0.82, the correlation coefficient for the test-retest was 0.92 (P＜0.001), and the intraclass coefficient for interrater reliability was 0.96 (95% confidence interval [CI] 0.90-0.99).

1. The University of California, Los Angeles Loneliness Scale, version 3 (UCLA-LS3):

The University of California, Los Angeles Loneliness Scale, version 3 (UCLA-LS3), is a revised version of the original version of the UCLA-LS by Russell. This revised scale has been adapted and validated in various subjects across different countries. A 3-item version (SF-3), based on the 20-item multidimensional revised UCLA-LS, was developed by Hughes. The three items on the SF-3 were selected because they showed the highest loading on the first factor of a three-factor model. The alpha coefficient for reliability was 0.72.

1. The Philadelphia Geriatric Center (PGC) Morale Scale

A model containing 11 of the original the Philadelphia Geriatric Center (PGC) Morale Scale items adequately fit both the American and Japanese data.

Based on your feedback, we examined the reliability of the outcome variable and added the finding to the Results (p. 10, lines 225–228).

Regarding the financial aspect, we considered whether we would ask participants about their current income/savings and expenditures. However, we anticipated low response rates, reasoning that some older adults may be reluctant to answer questions relating to their personal information or may find it difficult to answer such questions. We also considered that using a large number of scales would be physically and mentally taxing for older adults. Therefore, in this study, we asked participants about their subjective sense of anxiety about their economic situation. Specifically, to determine their sense of economic insecurity, we asked them to respond to the question, “Do you have economic anxieties?” using a four-point Likert scale (not worried, not too worried, a little worried, worried) (p. 6, lines 123–132).

8. Line 151: UCLA: Mention the full name

**Reply:**

Thank you for your comment. We added the full name for UCLA (The University of California, Los Angeles) (p. 7, lines 160–161).

9. Excluding 155 participants from the analysis may introduce bias. Did you consider attrition analysis? Did you consider missing data analysis (such as, MCAR test)? Why did you consider missing data replacement?

**Reply:**

Thank you for these insights. We compared and analyzed the 155 participants who were excluded from the analysis with those who were included in the analysis (Supporting information, Table. Comparison between Analyzed and Excluded Participants). These results were added to the Discussion (p. 27, lines 330–343) and Research limitations (p. 29–30, lines 400–404) sections.

Because the data in this study were not normally distributed, missing data analyses such as MCAR were not performed.

Among all participants included in the study, social activity participation was unknown for 64 participants and missing answers for basic attributes (e.g., gender, age) and illness were observed for 46 participants; these data were excluded from the analysis. In addition, 45 participants with a deficit of 5% or more in each scale were excluded from the study because of bias introduced by assigning mean values, etc. (Reference 30). (p. 8 lines 173–176).

10. Please consider drawing a flow chart that show the number of participants at each stage recruitment, dropout, etc.)

**Reply:**

　　　Thank you for this suggestion. We accordingly developed a flowchart of the participants in the study (Figure 1).

11. What about sample size calculation?

**Reply:**

Thank you for this question. The sample size for this study was analyzed using G*Power. Based on statistical power calculations, the sample size was 602. We added this information to the Analysis Methods section and cited relevant articles (p. 5, lines 102–106).

12. Table 2: It is unclear why you stratified the sample by age group. Please explain in the Method section.

**Reply:**

Thank you for this query. Life expectancy is increasing globally, and the number of older adults aged 85 and over is accordingly growing. In response to your excellent suggestion, we added our rationale for stratifying our sample by age group to the Analysis Methods section; this decision was based on prior research (Reference 31) (p. 8, lines 176–181).

13. Table 2: Please compute the effect size (p-value is not enough)

**Reply:**

Thank you for this suggestion. We computed the effect sizes and included them in Table 2 (p. 12–16, lines 254–259).

14. Table 3: Please compute the effect size (p-value is not enough)

**Reply:**

Thank you for this guidance. As above, we computed the effect sizes and included them in Table 3 (p. 18–22, lines 273–277).

15. Data reported in Table 1 is already reported again in Tables 2 and 3 (please delete any duplication and consider combining tables together).

**Reply:**

Thank you for pointing this out. We combined the tables and removed the table that was originally Table 1.

16. Only 10% of the participants use LTC insurance. Does this mean that the overall condition of the study participants

is relatively good, which may affect their response? I am curious to what extent the current sample represent the community-dwelling older adults in Hiroshima (or in Japan). This might hinder the generalizability of the current results.

**Reply:**

Thank you for this question. The older adults in the study were relatively physically and cognitively independent and able to complete the self-administered questionnaire survey and participate in social activities. According to a report on the implementation of the long-term care insurance system in Hiroshima Prefecture, in 2022, the percentage of people certified as having a low long-term care insurance level (support needs: levels 1 and 2, and care needs level 1) in Japan was 9.3% (10.1% in Hiroshima Prefecture), which was similar to the 10.7% rate in this study. We added this information to the Discussion (p. 26, lines 308–316) and included more References to substantiate these claims (Reference 34–36).

17. Table 3: the column "Category" is unclear. For example, in "Divorce," are you reporting data for "yes" or "no"?

**Reply:**

Thank you for checking in about this. We have revised Table 3 to clarify this point (we are reporting data for yes/no responses). Additionally, we modified our results to more closely report the results of the logistic analysis by age group (p. 23–25, lines 280–304).

18. Table 3: Did you examine multicollinearity?

**Reply:**

Thank you for this query. We added a discussion of multicollinearity to the Results (p. 23, lines 283–284;

p. 23, 288–289 and 295–296).

19. In the Discussion section, please do not re-write the results again (see for example Line 304).

**Reply:**

Thank you for this insight. We agree and have incorporated this suggestion in our revised manuscript.

20. Add a sub-section for the Implication.

**Reply:**

Thank you very much for this guidance. We accordingly added an Implication subsection (p. 29, lines 388–392). The implication of this study is that health professionals who promote community-based social participation can provide support tailored to older adults' specific ages and levels of psychological and economic insecurity. This can improve older adults’ quality of life, including their mental health, and prevent frailty.

Dear. Reviewer #2

Thank you for giving us the opportunity to strengthen our manuscript with your valuable comments and queries.

The study investigates the factors associated with the sense of economic insecurity in a select group of older people consisting predominantly of females who participate in government sponsored social activities. The main findings were that the sense of economic insecurity was associated with the age cohort 75-84 years compared with > 84 or 66-74years, increasing loneliness and with subjective sense of well-being (inverse association).

Although the overall study design and analysis are appropriate, the authors may wish to address the following:

1) The generalizability of the findings of this study is limited as the sample was derived form a select group that attends social activities. And I believe that this should be adequately emphasized as one of the major limitations.

**Reply:**

Thank you very much for your excellent suggestion. We agree. As you pointed out, the study population was rather specific; it comprised older adults who were relatively physically and cognitively independent and who were able to complete a self-administered questionnaire survey and participate in social activities. In response, we highlighted the limited generalizability of the findings in the Discussion (p. 26, lines 308–316; p. 27, lines 330–343) and Limitations (p. 29–30, lines 400–404).

2) To my understanding, although the authors have explained the rationale for selecting covariates in model 1, they have not done so with respect to models 2 and 3, and some of these variables have collinearity, for example loneliness, social isolation and marital status – perhaps authors may want to comment on this.

**Reply:**

Thank you for your suggestion. We added our rationale for the selection of the covariates in Models 2 and 3 and detailed the multicollinearity in the Results (p. 23, lines 284–296).

3) Some sections under results seem redundant, for example the authors have provided a fairly detailed account of the baseline sample characteristics in tables (table2) and text. I’m not sure such details on sample characteristics are necessary as they are not relevant to the objectives of this study.

**Reply:**

Thank you for these insights. Life expectancy is increasing globally, and the number of older adults aged 85 and over is accordingly growing. In response to your excellent suggestion, we added our rationale for stratifying our sample by age group to the Analysis Methods section; this decision was based on prior research (Reference 31) (p. 8, lines 176–181). To remove the redundancies in the results section, we have combined the tables and removed what was originally Table 1.

4) In the introductory section, the authors state “For low-cost social activities to function as a place for preventing frailty and suicide, it is necessary to investigate the actual state of the participants’ economic insecurity and discuss how to provide support for effective social activities” (lines 86–88) are there any references or data to back up this statement.

**Reply:**

Thank you for checking in about this. Prior research reported that community-level social participation and support prevent suicide (Reference 15). De Leo reported that suicide prevention for old adults should focus on the number of socio-environmental conditions that can be particularly worrisome in old age, such as decreased physical health, social isolation and loneliness, and economic insecurity (Reference 16). We added new text and references to the Introduction to substantiate these claims (p. 4, lines 82–86).

5) The authors state that the findings of this study are somewhat different from previous studies in that no association was found between physical, cognitive or social aspects and economic insecurity. However, I don’t think that the authors have discussed the likely reasons for these observed differences.

**Reply:**

Thank you very much for this observation. In response, we included some possible reasons for these differences in the Discussion section (Reference 38) (p. 26–27, lines 324–343).

6) I believe that the authors have to be circumspect with respect to some of assertions they have made in the discussion. For example, the authors state (lines 329–334 “in this study psychological conditions were associated with a sense of economic insecurity among older adults, highlighting the importance of preventing early frailty progression through social activities. Although the study findings support the 1st part of this statement, viz, in this study psychological conditions were associated with a sense of economic insecurity among older adults, their comments about preventing frailty cannot be inferred from the findings. Similarly, their basis for stating “to prevent loneliness and improve subjective well-being, establishing places where older adults-----------------social activities at low cost will improve their quality of life is not supported by the findings of this study.

**Reply:**

Thank you for this feedback. In this study showed that psychological status was associated with economic insecurity among older adults. As your comment, we cannot infer from the results of this study regarding the prevention of frailty. We agree with your assessment and have made according changes to the Discussion (p. 29, lines 384–387) and the Abstract (p. 2, lines 47–49). Notably, we added a reference (Reference 45-47) in our revisions based on your comment to the Discussion. We also highlighted that a key implication of this study is that social participation can prevent frailty (p. 29, lines 388–392).
