# Supplementary material for: Factors related to a sense of economic insecurity among older adults who participate in social activities
Source: PLoS One. 2024 Mar 28;19(3):e0301280. doi: 10.1371/journal.pone.0301280 (PMC10977778; doi:10.1371/journal.pone.0301280)
Supplement: Attachment — Submitted filename: renamed_bf6c6.docx [file pone.0301280.s004.docx]

Responses to Reviewers’ Comments

Manuscript Number：PONE-D-23-12908R1

Full Title: Factors related to a sense of economic insecurity among older adults who participate in social activities

Dear. Reviewer #2

Thank you for giving us the opportunity to strengthen our manuscript with your valuable comments and queries.

1) The references 15 and 16 in the introduction, the authors have now cited in their response, refer to the association between environmental factors /activities and suicide or suicide prevention, and are not relevant frailty. Therefore, they are not the appropriate references for suggesting any potential association between the sense of economic insecurity or social activities and frailty.

**Reply:**

Thank you for pointing this out. In response to your suggestion, we removed Reference 15 because it was an article that referred to the association between environmental factors/activities and suicide or suicide prevention. Reference 16 has been moved to the section related to suicide in the Introduction (accordingly, Reference 16 is Reference 9 in the revised manuscript) (p. 3, lines 69–72). Additionally, we have added references that demonstrate any potential association between the sense of economic insecurity or social activities and frailty (References 16 and 17) (p. 4, lines 86–91).

2) The authors now mention the sample size but fail to discuss estimated power or set alpha values.

**Reply:**

Thank you for this suggestion. We set a power of 0.80 and an alpha value of 0.05. We have added this information to the Research participants subsection of the Methods section (p. 5, line 108).

3) The authors may wish to consider sensitivity analyses including the 155 participants excluded from analysis, as there are significant differences in their baseline characteristics - they are significantly older, more likely to have dementia symptoms and use long-term care insurance.

**Reply:**

Thank you for your feedback. The 155 excluded individuals were willing to participate, but were excluded from the analysis because they did not respond to certain items, such as missing 5% or more on each scale or items regarding participation in social activities. They may have found it difficult to answer owing to some influence on their physical, mental, or cognitive functions. This shows that people with dementia symptoms and older adults using long-term care insurance also participate in social activities at the community level. We believe that it is important for older adults to participate in social activities that provide them with opportunities to access a variety of information and to meet kindred spirits in order to prevent the progression of frailty. In future studies, it is necessary to obtain the cooperation of the community general support center staff to expand the number of participants so that all those who participate in social activities can be analyzed. We have added the underlined section to the Discussion (p. 27, lines 345–355).

4) Under implications, the authors state “ Regarding the implication of this study, it reveals that health professionals who promote community- based social participation can provide support tailored to older adults’ age and level of psychological and economic insecurity . This work can improve older adults’ quality of life , including their mental health, and prevent frailty”. I’m not sure one could draw these conclusions as they are not underpinned by the findings of this study – the study did not investigate the potential consequences or implications of the sense of economic security, or the possible interventions to ameliorate the sense of economic security. Similarly, the concluding section too was outside the scope of the findings of this study. For example, I don’t believe that the findings of this study support the following conclusion – “Notably, this study showed that preventive measures to limit loneliness and increase subjective well-being through the use of social activities play an important role in reducing economic insecurity”.

**Reply:**

Thank you for these insights. We agree. As you pointed out, the design of this study is cross-sectional, and further longitudinal and intervention studies are needed in the future to verify the associations. In response, we have revised and deleted the parts of the Implication (p. 29, lines 402–404), Limitations (p. 30, lines 412–414), and Conclusions (“Notably, this study showed that preventive measures to limit loneliness and increase subjective well-being through the use of social activities play an important role in reducing economic insecurity”.) that were pointed out by you.
