# Supplementary material for: Factors related to a sense of economic insecurity among older adults who participate in social activities
Source: PLoS One. 2024 Mar 28;19(3):e0301280. doi: 10.1371/journal.pone.0301280 (PMC10977778; doi:10.1371/journal.pone.0301280)
Supplement: Attachment — Submitted filename: Responses to Reviewers.docx [file pone.0301280.s005.docx]

Responses to Reviewers’ Comments

Manuscript Number：PONE-D-23-12908R2

Full Title：Factors related to a sense of economic insecurity among older adults who participate in social activities

Dear. Editor

Thank you for giving us the opportunity to revise and strengthen our manuscript based on your valuable comments and queries.

1) The sample size calculation is unclear. The mentioned description is insufficient. You need to describe in detail how the sample size was computed including the name of the software used.

**Reply:**

Thank you for your comment. We used G*Power to calculate our sample size. The Cabinet Office 2020 White Paper on Older Adults reports that 74.1%, or three-quarters of those aged 60 and older, are living without economic insecurity, more than that in the 2016 survey (see Annual Report on the Ageing Society [Summary] FY2020: https://www8.cao.go.jp/kourei/english/annualreport/2020/pdf/2020.pdf). However, we expected the percentage of older adults with economic insecurity to increase to approximately 30% with the COVID-19 pandemic. We predicted that those with good physical, social, and psychological status would be 0.8 times more likely to have economic insecurity than those with low economic insecurity, which we set at 602 on a one-tailed test. We also examined the sample size based on the Events per variable following Peduzzi et al., who reported that data should be collected so that the Events per variable is greater than 10 (Peduzzi et.al., 1996). We examined the following explanatory variables to identify the factors associated with economic insecurity: basic attributes: 1) gender, 2) age, 3) household composition, 4) marital status, spouse status (5) bereaved, 6) divorced, 7) separated), 8) whether the participant had a child (children); physical conditions:9) presence of illness, 10) subjective symptoms of dementia, 11) whether long-term care insurance services were used; social conditions:12) frequency of outings, 13) frequency of participation in social activities, 14) working status, 15) the presence or absence of social isolation; and psychological conditions: 16) loneliness, and 17) subjective well-being. Assuming 30% of the respondents have a sense of economic insecurity, the sample size must be at least 567.

2) In the STATISTICAL ANALYSIS section, p-value is on-tailed or two tailed?

**Reply:**

Thank you for your concern. We performed a two-tailed test. We have added the following sentence to the manuscript to reflect this: “A two-tailed p-value of <0.05 was considered to indicate statistical significance.” (p. 9, lines 212-213).

3) Table 1: you used symbols a,b and A,B,C. This is confusing. Do not use capital and small forms of the same letter to denote different things. Please use different symbols. Also, reporting the results of the post-hoc analysis (A,B,C) is unclear. You may need to consult a statistician about how to report the results of the post-hoc analysis clearly. For example, what is the meaning of BC and ABC?

**Reply:**

Thank you for this suggestion. We have revised the symbols “a” to “†” and “b” to “‡”. We have also corrected the reporting of the post-hoc analysis results to “†: χ² and z-tests were performed to compare column proportions. p-values have been Bonferroni corrected. a: vs 65-74 years old, b: vs 75-84 years old, p<0.05” (p. 12-16, lines 259-262).

References:

1) Mielgo-Ayuso J, Aparicio-Ugarriza R, Castillo A, Ruiz E, Ávila JM, Aranceta-Batrina J, et al. Physical activity patterns of the Spanish population are mostly determined by sex and age: Findings in the ANIBES study. PLoS One. 2016;11(2):e0149969. doi: 10.1371/journal.pone.0149969.

2) Koyama A, Hashimoto M, Tanaka H, Fujise N, Matsushita M, Miyagawa Y, et al. Malnutrition in Alzheimer’s disease, dementia with Lewy bodies, and frontotemporal lobar degeneration: Comparison using serum albumin, total protein, and hemoglobin level. PLoS One. 2016;11(6):e0157053. doi: 10.1371/journal.pone.0157053.

4) You need to elaborate on the IMPLICATIONS of the current study (Line 401).

**Reply:**

Thank you very much for this suggestion. The implications were suggested by Reviewer #1, and in the second draft, the following text was added: “Regarding the implication of this study, it reveals that health professionals who promote community-based social participation can provide support tailored to older adults' age and levels of psychological and This work can improve older adults' quality of life, including their mental health, and prevent frailty.” In the third draft, we revised parts of the text in accordance with the suggestions we received from reviewer #2 as follows: “Regarding the implications of this study, our results may lead health care providers who promote community-based social participation to consider support tailored to older adults’ age and psychological and economic insecurities.” We agree with your suggestion to elaborate on the implications, so we have further revised the paper in Draft 4 as follows: “Regarding the implications of this study, our results may lead healthcare providers who promote community-based social participation to consider support tailored to older adults’ age and psychological and economic insecurities. Furthermore, given that nearly half of the older adults participating in social activities felt economic insecurity, receiving support to alleviate this issue through social activities could contribute to improving their quality of life, including their mental health.” (p. 29, lines 401–407).

5) Figure 1: The quality is extremely low. Please provide a hig-quality figure.

**Reply:**

Following on your suggestion, we have re-created Figure 1 to ensure that it is of high-quality.
